# 

**Published:** 1895-10-05

**Authors:** 


### The Hospital

AN INSTITUTIONAL JOURNAL OF
THE MEDICAL SCIENCES AND HOSPITAL ADMINISTRATION,

INCLUDING

A SPECIAL SUPPLEMENT FOR NURSES.

EDITED BY

HENRY C. BURDETT.

IN TWO VOLUMES ANNUALLY.

SURGEON GENL'S OFFICE

154376.

LIBRARY

VOLUME XIX.

October, 1895, to March, 1896.

London:
PUBLISHED BY THE SCIENTIFIC PRESS (LIMITED), AT THEIR OFFICE, 428, STRAND, W.C.

MDCCCXCVI.

W1 No69X

### INDEX TO VOL. XIX., 1895-96.

*** Articles under the general heading *Medical Progress and Hospital Clinics* are distinguished by the initial (C.) for *Hospital Clinics*, and by (P.) for the rest of the series. (W.) distinguishes the *Institutional Workshop* articles, and (A.H.) the notes under the general title *Around the Hospitals.*

Abdomen, Traumatic Lesions of the (P.), 331
Abdominal Contents and Tight Lacing. See p. 165
Abdominal Operations, The Treatment of Patients after (C.), 7, 27
Abdominal Section in Puerperal Peritonitis (P.), 101
Abdominal Surgery (P.), 331
Abdominal Tumours, Disappearing (P.), 399
Abnormality or Madness, 77
Abscess, Chronic Spinal (P.), 269
Abscess, Hepatic (P.), 30
Abscess in Pott's Disease, Treatment of (C.), 79, 97 (P.), 332
Abscess in Pott's Disease, Absorption of (C.), 80
Abscess, Parapancreatic, 232
Abscess, Subphrenic (P.), 252
Accrington District Cottage Hospital (W.), 404
Achylia Gastrica (P.), 382
Acne (P.), 432
Acromegaly and Graves' Disease (P.), 80
Actinomycosis (P.), 251
Addresses, Introductory, at the Medical Schools, 6
Adulterated and Diseased Foods (P.), 167
Albuminuria (P.), 348, *and see p.* 220
Alcohol and Longevity, 323
Alcohol Question, The (P.), 166
Anæmia Leukæmia and Lymphadenoma (P.), 200
Anæsthetics, The Administration of, 75, 143
Anatomy, Topographical (P.), 331
Anchylostoma Duodenale (P.), 416
Andrew Clark Memorial, The, 209 *and see p.* 32
Aneurism, Diagnosis of, 57, 111
Aneurism, The Treatment of, 211, 309
Aneurisms, On, 425
Angio-Keratoma of the Right Vocal Cord (P.), 50
Angiocholitis and Cholecystitis (P.), 417
Ankylosis (P.), 334
Annus Medicus, 1895, 225
Anthropology, 23; Practical, 127; Education in, 195
Anthropology at the Universities, 302
Antiseptics (P.), 184
Anti-Vivisection Societies and the Hospitals, The, 154
Apolyrin (P.), 400
Appeal to Appealers, An, 74
Appendicitis (P), 99, 431
Arecoline, Bromhydrate of (P.), 269
Argonine and Argentamine (P.), 13
Around the Hospitals (A. H.) *See also under the names of the various hospitals*, 36, 88, 190, 288
Arthrodesis (P.), 333
Aspergillus Flavescens in the Auditory Canal (P.), 134
Aspiration (in Potts' Disease) (C.), 97
Bacteriology and Pathology (Lung Disease) (P.), 282
Basedow's Disease, Incipient (P.), 49
Bath Royal Mineral Water Hospital (A. H.), 88
Beri Beri (P.), 251
Billings, Dr. John S., 40
Birmingham Eye Hospital (W.), 240
Bladder, Hernia of the (P.), 132
Bladder, Tuberculous Disease of the (P.), 117
Bladder, Tumours of the (P.), 132
Board School Teachers, Vocal Break-down on the part of (P.), 65
Book World of Medicine, The (*Books, Magazines, and Pamphlets reviewed or noticed, and articles relating to*):—
Barnard and Stocker. Medical Partnerships, Transfers, and Assistantships, 70
Beale. *See Harris*
Burdett. Cottage Hospitals: General, Fever, and Convalescent, 353
Burford, Shaw, and Moir. London Homœopathic Hospital Reports, 273
Carter. Elements of Practical Medicine, 223
Clerke. The Herschells and Modern Astronomy, 89
Davis. Elementary Physiology, 208
Ferrero. *See Lombroso*
Ferri. Criminal Sociology, 305
Firth. *See Notter*
Forward. The Case against Butchers' Meat, 70
Foxwell. Essays in Heart and Lung Disease, 191
Garden Oracle, &c., The, 405
Gillies. Theory and Practice of Counter-irritation, 107
Green. Manual of Botany, 241
Harris and Beale. Treatment of Pulmonary Consumption, 405
Hart. Diet in Sickness and Health, 207
Keith. A Plea for a Simpler Life, 196
Kelly's London Medical Directory 1896, 371
Lombroso and Ferrero (Intro. by Morrison). The Female Offender, 4
London Homœopathic Hospital Reports, 273
MacDowall. Weather and Disease, 89
Marey (trans. by Pritchard). Movement, 278
Medical Annual 1896, The, 439
Moir. *See Burford*
Moor. *See Permain*
Morrison. *See Lombroso*
Book World of Medicine, The—(*continued*).
Neave. Medical Digest, 405
Notter and Firth. Hygiene, 305
Ouida. Toxin, 273
Our Treasures, and how to keep them, 337
Partridge. Practical Ambulance Tablets, 223
Pedley. Diseases of Children's Teeth, 289
Permain and Moor. Aids to the Analysis of Food and Drugs, 241
Pritchard. *See Marey*
Roberts. Modern Medicine and Homœopathy, 371
Science Progress, 305, 371
Sexton. Inorganic Chemistry, 353
Shaw. *See Burford*
Sleman, The Volunteer Surgeon's Guide, 19
Smyth. A Baneful Popular Delusion on the Subject of Motherhood, 107
Snow. Cancers and Tumours, 89.
Stacpoole. A Household Guide for Use in Sickness, 19
Stewart. Manual of Physiology, 191
Stocker. *See Barnard*
Smith. A Medical and Surgical Help for Shipmasters, &c., 70
Tallack. Penological and Preventive Principles, 310, 376
Tallerman. Sheffield Patent Localised Hot-air Bath, 289
Thompson. The Chemists' Compendium, 337
Thornton. Memories of Seven Campaigns, 70
Twentieth Century Practice of Medicine, 19
Williams. Diseases of the Upper Respiratory Tract, 157
Wright. Gout and Rheumatic Gout, 337
Year Book of Treatment for 1896, 371
Boots, The Dangers of Adulterated, 113, *and see p.* 236
Boston City Hospital Tent Service, 272
Bovine Tuberculosis, Some Practical Aspects of, 91
Brain Surgery (P.), 10
Bread (P.), 167
Breast, Tumours of the (P.), 203
Breasts, Care of the, After Labour (P.), 133
British Army as a Fighting Force, The, 340
British Balneological and Climatological Society, 286
British Medical Association and Quackery, The, 247
British Medical Association, The Discontent in the, 423
Bromoform (in Lung Disease) (P.), 267
Bulbar Crises (P.), 348
Burton-on-Trent, New Hospital for (W.), 288
Butcher, Down with the, 196
Butter to Brains, From, 295
Cancer (P.), 383
Cancer-stricken, a Gleam of Hope for the, 95
Carbohydrate Starvation in Typhoid Fever, 342
Cardiac Murmurs. *See p.* 228
Carrying Chair. *See pp.* 155, 186.
Caxton Convalescent Home, Limpsfield (W.), 351
Cerebral Surgery (P.), 300
Cerebral Tumours (P.), 317; Operations for, 10
Cerebro-Spinal Meningitis (P.), 251
Chalfont St. Peter Epileptic Home (W.), 172
Charity and the Press, 213
Charity or Business. *See pp.* 264 and 321
Charity Organisation Society (W.), 171
Chelsea Hospital for Women, The, 197, 213
Chest, Surgery of the (P.), 251
Chlorosis, A Latent Danger in, 263
Cholecystitis and Angiocholitis (P.), 418
Choledocho-Duodenostomy, Internal (P.), 418
Cholelithiasis (P.), 29, 417
Cholera (P.), 203
CHRISTMAS APPEAL SUPPLEMENT:—
H.R.H. the Prince of Wales, 13
A Merry Christmas for Once at the Hospitals, 15
Choosing a Charity, 15
Gay with Flowers, 16
Medical Student of To-day, The, 17
Dinner Hour, The, 18
What the Press is Doing for Christmas, 19
Sweet Charity's Guide to Christmas Givers, 20
Citrophene and Apolyrin—New Anti-pyretics and Anti-neuralgics (P.), 400.
Clark Memorial, The Sir Andrew, 32, 209
Climates of the Past, 144
Climatic Treatment of Lung Disease (P.), 267, 282
Clinical Recollections, 293, 391
Clinical Society, 350
Clinics of the Future, The, 179
Clothes Philosophy, a Prophylactic, 307
Club-foot (P.), 333
Colds, A Novel Cure for, 343
Colitis (P.), 100
Colliery Disaster, The, 292
Colour-blind Sailors, Fate of, 77, *and see p.* 102
Commissariat—Hospital Expenditure (W.), 15, 33, 51, 85, 103, 121, 137, 153, 187, 205, 401, 419, 435
Competition, Medical, The Limits of, 275
Congresses, The Three, Their Lessons to Medicine, 39
Construction Notes (W.), 17, 36, 68, 88, 104, 190, 239, 257, 304, 351, 404
Consumption and Dyspepsia, 325, 357, 409
Convalescent Homes—Dunoon (W.), 287; Limpsfield (W.), 351
Coolgardie, The General Hospital (A. H.), 190
"Coromandel, The," Hospital Ship (W.), 188
Coroner, The Proper Business of the, 193
Coroners and Practitioners, 276; *and see p.* 350
Cottage Hospitals (W.). *See pp.* 104, 239, 257, 303, 371, 404.
Cranial Meningocele (P.), 269
Cranial Tension. *See Intra-Cranial Tension*
Craniectomy (P.), 317
Creusot, Hôtel-Dieu du (W.), 221.
Crime. Is Crime Decreasing? 392
Criminals, The Treatment of, 310
Cruel "Surgical Operation," A, 359
Curette, Use of the, in cases of Puerperal Infection (P.), 101
"Current that Kills, The," 43
Cycling, The Dangers of Street, 25
Danger to the Medical Profession, A, 343
Dead Weight of the Sick, The, 291
Deaf, Oral Instruction of the (P.), 120
Deaf Mutes and the School Board, 427
Deafness. Discussion on Nerve Deafness at the B.M.A. (P.), 134
Deafness, Etiology of (P.), 119
Death Certificates, 350
Definition, An Injudicious, 359
Derbyshire Royal Infirmary (A. H.), 288
Dermatitis Repens (P.), 434
Dermatology, Periscope of (P.), 432
Dermoid Cyst (P.), 10
Diabetes and Graves' Disease (P.), 80
Diarrhœa, Infantile and Artificial Feeding, 374
Digestive Organs, Diseases of the (P.), 99
Digestive Organs, Minor Ailments of the (P.), 101
Diphtheria Treated with Ruffer's Curative Serum (C.), 215
Diphtheritic Neuritis (P.), 347
Discretionary Bequests (W.), 335
Disinfecting Machine, A New French (W.), 222
Disinfection and the Existing Data for its Practice (W.), 16, 34, 86, *and see p.* 106
Dislocation (Congenital) of the Hip and of the Shoulder (P.), 333
Dislocation of the Peroneus Longus Tendon (P.), 333
Dislocation. *See Fractures.*
Doctors *versus* Drink, 160
Drunk or Dying, 197
Dunoon Convalescent Homes (W.), 287
Duodenum, The (Diseases of the Digestive Organs) (P.), 100
Dysentery (P.), 100
Dyspepsia and Consumption, 325, 357, 409
Ear. *See Otology and Middle Ear*
Early Marriages, Royal and Otherwise, 24
Easter, The Coming of, 424
Edinburgh, New Royal Hospital for Sick Children (W.), 104
Editor's Letter Box, 18, 102, 136, 156, 186, 236, 272, 321, 350, 371, 418, 439
Electricity and the Processes of Life, 92
Empyæma, The Mode of Healing of (P.), 252
Energy, The New Radiant, 308
Epidemics, The Prevention of, 229
Epilepsy (P.), 379, The Treatment of, 177 (P.), 381
Epilepsy, Idiopathic, Excision of Cortical Areas in (P.), 301
Epileptic Colony at Chalfont St. Peter (W.), 172
Epileptics in America (*note*), 425
Erysipelas (P.), 433
Erysipelas Toxins and Serum in Malignant Growths (P.), 203
Ethics of Operations, The, 373
Examinations of the Future, The, 21
Families, Large or Small? 43
Farnham Cottage Hospital, Trimmer's (W.), 257, 303, 371
Femoral Hernia, On the Treatment of (C.), 413
Femur, Incurvation of the Neck of the, in Adolescence (P.), 333
Fever Hospital, How to Get into a, 179
Fevers, The Pathology of (P.), 182
Fifteen Reasons for Vaccination, 327
Fifteen Thousand Pounds for the Charities, 74
Fighting, The Physiology of, 259
Fistula. *See p.* 115
Fistula in Ano, Treatment of, by Scraping and Suture (P.), 268
Flat-foot (P.), 333
Flat-foot, Acquired, The Treatment of (C.), 429
Floating Hospital on the River Tees (P.), 255
Fracture, Un-united, Causes and Treatment of (C), 61.
Fractures and Dislocations (P), 150, 163, 183
Fractures of the Patella, The Treatment of (C.), 329, 345
Frontal Sinus, Distension of the (P.), 300
Furniture and Fittings for Nurses' Homes (W.), 52, 188, and *see p.* 288

Furuncle of the Meatus, A New Mode of Treating (P.), 135
Gall Bladder, Perforation of the, following Typhoid Fever (P.), 31
Gastric Catarrh (P.), 382
Gastric Crises (Movable Kidney) (P.), 233
Gastric Ulcer, Perforation of a (P.), 399
Gastrotomy (P.), 399
General Medical Council, 170
General Pathology (P.), 182, 202
General Surgery (P.), 150, 168, 183
Genito-urinary Surgery (P.), 116, 132, 185
"Gorham" Bed, The (W.), 271
Gout and Rheumatism (P.), 315
Graves' Disease (P), 99; Pathology of (P), 62, 80; Operative Treatment (P.), 81
Greenwich, Miller Hospital (A.H.), 88
Growths, Morbid (P.), 203
Guy's and Mr. Gladstone's Appeal, 77
Hæmato-porphyrin. *See p.* 219
Hæmic and Organic Cardiac Murmurs, The Relation between, 228
Hæmorrhage, Post-partum, A Rare Form of (P.), 133
Hæmorrhoids—Causes and Symptoms (C.), 218, 297
Hammer-toe (P.), 334
Harveian Oration, The, 59
Head Injuries (P.), 283
Heart Disease, Physical Treatment in (C.), 298, 349, (P.) 318
Heart Disease, Schott Treatment of (C.), 46, 249
Health Work of a City Company, 163
Helpers or Rivals? The Nurse Question, 339
Hepatic Abscess (P.), 30
Heredity and the Race, 178
Hernia (P.), 363-366
Hernia, Femoral (C.), 413
Herniæ, Vesical (P.), 364
Hero, A Forgotten Medical, 112
Heterophoria, 341
High Feeding, In Praise of, 212
Hip, Congenital Dislocation of the (P.), 333
Holloway Sanatorium, The, 159
Homeless Ones, Our, 113
HOSPITAL, THE, A Medical Journal, 1
Hospital Abuse, One Cause of, 377
Hospital Committees and Hospital Secretaries (W.), 237
Hospital Construction (W.), 53, 104, 171, 287, 303, 368, 436
Hospital Expenditure (W.), 15, 33, 51, 85, 103, 121, 137, 153, 187, 205, 401, 419, 435
Hospital Festivals and Meetings &c. (W.), 17, 87, 106, 138, 155, 172, 189, 206, 238, 402, 419, 437
Hospital Meetings. *See Hospital Festivals*
Hospital Saturday. Why Hospital Saturday Fails in London, 229
Hospital Secretaries and their Salaries (W.), 257, *and see p.* 272
Hospital Ship, "The Coromandel" (W.), 188
Hospital Sunday Fund, Metropolitan, 1895, 67, 86, 105, 122, 155, 319
Hour-glass Contraction of the Stomach (P.), 399
Hull, A Sanitary Record for, 179
Hunger as a Test of Poverty, 110
Huxley, A Statue for Professor, 129
Hydatid of the Liver, Suppurating (P.), 417
Hyperacidity (Gastric Affections) (P.), 9
Hyperchlorhydria (P.), 382
Hypertrophy, Moriform, of the Inferior Turbinated Bodies (P.), 66
Hypertrophy, Prostatic (P.), 116, Turbinal, (P.), 66
Hypodermic Feeding (P.), 167
Iceland, A Hospital for Lepers in (W.), 221
Icthyol (in Lung Disease) (P.), 267
Idiopathic Epilepsy, Excision of Cortical Areas in (P.), 301
Incision and Drainage (Pott's Disease) (C.), 97
Infantile Diarrhœa and Artificial Feeding, 374
Infection, Latency of, 243
Infectious Diseases, Private Hospital for, New York (W.), 368
Infective Diseases (P.), 250, 299
Influence of Mind upon Body, The, 324
Influenza (P.), 299
Insane: Can the Treatment of the Insane be Improved? 176
Insane: Story of the Insane from Year to Year, 35, 68, 206, 237, 271, 368
Insanity, Pathology of (P.), 396
Intestinal Antisepsis (P.), 416
Intestinal Occlusion after Laparotomies (P), 332
Intestines, Diseases of the (P.), 416
Intra-Cranial Tension, Operations for the Relief of (P.), 316
Ipecacuanha, Alkaloids of (P.), 384
Ischio-rectal Abscess and Fistula (C.), 115, 131
Jameson (Dr.) and South African Medicine, 427
Kidney, Movable (P.), 233
Knavery Extirpation Society, A, 213
Knee Jerks (Neuritis) (P.), 348
Laryngitis, Chronic (P.), 65
Laryngology (P.), 12, 31, 49, 65
Larynx, An Artificial (P.), 49
Larynx, Benign Growths of the (P.), 50
Larynx, Total Extirpation of, for Malignant Disease (P.), 50
Larynx: Pulsus Paradoxus in Acute Laryngitis (P), 50
Larynx, Tubercular Tumours of the (P.), 50
Laundry and Sanitary Exhibition at the Agricultural Hall, 138
Lepers, A Hospital for, in Iceland (W.), 221
Lesions of the Abdomen, Traumatic (P.), 331
Lettsomian Lectures, The, 311
Leucocyte in Disease, The, 395
Leukæmia, Lymphadenoma, and Anæmia (P.), 200, 201
Limpsfield Convalescent Home (W.), 351
Liver, Rupture of the (P.), 417
Liver, Suppurating Hydatid of the (P.), 417
Lungs and Pleuræ, Diseases of (P.), 217, 231, 266, 282
Lupus (P.), 433
Lymphadenoma, Leukæmia, and Anæmia (P.), 200, 201
Malaria (P.), 203
Man, The Study of, 127, 195, *and see p.* 23
Mastoid Disease (P.), 300
Meatus, Furuncle of the (P.), 135
Median Incision, The (P.), 331
Medical Competition, The Limits of, 275
Medical Council and the Medical Profession, The, 163
Medical Department U.S. Army (W.), 336
Medical Men and Medical Women, 95
"Medical Temperance," 411
Medicine, A Stage in the Evolution of, 109
Medicine at Oxford, 358
Membrana Tympani, Rupture of the, from Violence (P.), 134
Meningitis, Cerebro-Spinal (P), 251; Tubercular (P.), 317
Meningococcie, Cranial (P.), 269
Mental Defects and Bodily Abnormalities, 377
Merycismus (P.), 8
Metatarsalgia, Morton's (P.), 333
Microbe, Fate of the, 263
Middle Ear, Cerebral Complications in Relation to the (P.), 135
Middle Ear, Inflammation of the, in Infants (P.), 135
Middle Ear, Septicæmia following Disease of the (P.), 152
Middle Ear, Tuberculous Disease of the (P.), 135
Milk as a Vehicle of Disease, 279
Morbid Growths (P), 203
Morphine and Opium (P.), 284
Morphine Poisoning, Antidotes in (P.), 284
Morphine Sickness (P.), 284
Mortal Calling, A, 113
Motion, The Mechanism of, 278
Movable Kidney (P), 233
Mucous Membrane, Vascular Mechanism of the Nasal (P.), 83
Municipal Dwellings for Working Men, 389
Murthly Asylum (W.), 240
Myxœdema and Graves' Disease (P.), 80
National Health, The Factors of, 355
Necrosis, Acute (C.), 361
Nephritis, Infectious (P.), 219
Nephrorraphy (P.), 233
Nerve Cells, Evolution of (P.), *see pp.* 414, 415
Nerve Centres, Frontal Lobes, Localisation of Activity in (P.), 415
Nerve Suture (P.), 234
Nerves, Surgery of the (P.), 11
Nerves The Repair of Divided (P.), 234
Nervous System, Diseases of the (P.), 379, 396, 414
Nervous System, Lesion of and Graves' Disease (P.), 81
Neural Infixation (P.), 285
Neuralgia, Trigeminal (P.), 235
Neurectomy of the Trigeminus, Intra-Cranial (P.), 11
Neuritis, Diphtheritic (P.), 347
Neuritis, Optic in Cerebral Tumours (P.), 10
Neuritis, Traumatic (P.), 235
New Appliances and Things Medical, 14, 32, 84, 120, 136, 152, 186, 204, 220, 235, 254, 270, 301, 318, 334, 350, 366, 384, 400, 418, 434
"New Child, The, as Made in Germany," 377
New York, Private Hospital for Infectious Diseases (W.), 368
Northallerton Cottage Hospital (W.), 239
Notes and News, 6, 25, 44, 60, 78, 96, 114, 130, 146, 164, 180, 198, 214, 230, 248, 264, 280, 296, 312, 328, 344, 360, 378, 394, 412, 428
Notification of Measles, The, 411
Nurse Question, The, 339
Nurses' Homes, Furniture and Fittings for (W.), 52, 188, *and see p.* 288
Obstetrics, Progress in (P.), 101, 133
Occlusions after Laparotomies, Intestinal (P.), 332
Œdema, Physiology of (C.), 45
Œdema, Angio-Neurotic (P.), 434
Œsophageal Pouch, Excision of an, 399
Œsophagus and Stomach, Surgery of (P.), 398
Œsophagus, Foreign Bodies in the (P.), 398
Œsophagus, Stricture of the (P.), 398
Omentum, A Rare Condition of the (P.), 332
Ophthalmic Medicine and Surgery, On Modern Progress in, 3, 41, 93, 161, 245, 261, 341, 375
Ophthalmology of the Poets, The, 326, 359
Opium Poisoning. *See p.* 285
Optic Neuritis in Cerebral Tumours (P.), 10
Orthopædic Surgery (P.), 332
Otitis Externa, Salicylic Acid in (P.), 135
Otitis Media Suppurativa, The Dry Treatment of (P.), 151
Otitis Media, Acute, Treatment of (P.), 135
Otology, Progress in (P.), 119, 134, 151
Out-patient System and its Abuse, The (W.), 367
Pancreas, Diseases of the (P.), 232
Pancreas, Rupture of the (P.), 232
Pancreatic Cysts (P.), 232
Pancreatitis, Acute (P.), 232
Papilloma Recurrent (P.), 50
Parapancreatic Abscess and Fat Necrosis (P.), 232
Parasitic Diseases (P.), 433
Parents' Point of View, The, 95
Pasteur, Louis, 2; The Death of, 5
Pasteurisation of Milk, The, 163
Patella, The Treatment of Fractures of the (C.), 329, 345
Patellar Tendon Reflex (P.), 415
Pathology, General (P.), 182, 202
Paupers, The Classification of, 5
Peat Wool as a Surgical Dressing, 152
Pemphigus (P.), 433
Penology, 376
Perineum, Preservation of the (P.), 133
Peritonitis, Puerperal. *See p.* 101
Permanganate of Potassium in Opium Poisoning (P.), 285
Perth, Murthly Asylum (W.), 240
Pharynx, Primary Tuberculosis of the (P.), 82
Philanthropists and the London Poor, 125
Photography, The New, in Court, 393
Phthisis, Relation of, to Insanity (P.), 397
Phthisis, The Use of Sedatives in, 277
Picric Acid as a Cure for Burns, 83
Pityriasis Rubra (P.), 434
Plaster Cast, The Horizontal Position for (Pott's Disease) (P.), 332
Pleuræ. *See Lungs.*
Pleural Effusion, Serous, The Treatment of (P.), 252
Pneumothorax (caused by a plumstone) (P.), 65
Poisoner, the Doctor, and the Nurse, The, 197
Poisoners, The Wholesale, of London, 145
Popliteal Swellings, On the Differential Diagnosis of (C.), 199
Porth Cottage Hospital (W.), 404
Post Partum Hæmorrhage, Rare Form of (P.), 133
Pott's Disease (P.), 332; Treatment of (C.), 79, 97
Practical Departments (W.), 16, 34, 52, 86, 138, 155, 188, 271, 304, 336
Practical Points, 106, 288
Prostatic Hypertrophy (P.), 116
Psoriasis (P.), 432
Public House and Sanitary Authorities, 411
Pylorus, Stenosis of the (P.), 9
Pyrexia (P.), 183
Rabies (P.), 299; The Extension of, 311
Radii, Congenital Absence of the (P.), 333
Rectum, Ulceration of the (P.), 268
Rectum, Vaginal Resection of the (P.), 269
Reformatory and Industrial Schools, Demoralising Tendencies of, 294
Reichmann's Disease (P.), 382
Relieving Officers and Medical Extras, 25
Remedies, Restrictions in the use of Unproved, 141
Renal Medicine (P.), 219
Research in Education, The Place of, 244
Resources of Civilisation, Some Unutilised, 73
Rheumatism, Gout and, 315, 316
Rheumatism, The Relation of, to Graves' Disease (P.), 81
Rhinology, Progress in (P), 66, 83
Rotunda Hospital, Dublin (W.), 173
Royal College of Physicians, The Presidency of the, 407
Ruffer's Curative Serum. *See p.* 215
Sac, Complete Removal of the (Pott's Disease) (C.), 98
Sac, The Coverings of the (Hernia) (P), 363
Salvation Army Shelters, 145, and *see p.* 142
Sanitation for Fishes—and Mayors, 229
Sanitation means Clean Living, 247
Sausage, Secrets of the Savoury, 395
Scarlatina (P.), 251
School Board Certificates, 194
Schott Treatment (C.), 47, 249
Scoliosis (P.), 333
Scraps and Gleanings, 18, 37, 53, 69, 88, 106, 124, 139, 156, 173, 190, 207, 223, 240, 257, 272, 289, 304, 337 404
Sequestra, Removal of (Pott's Disease) (C.), 98
Sewage in the Scotch Lochs, 56
Shanghai, The Health of, 43
Shoulder, Congenital Dislocation of the (P.), 333
Sick in Prisons, The Care of the, 426
Sinus, Frontal, Distension of (P), 300
Sinus, Superior Longitudinal, Primary Thrombosis of (P.), 301
Sixty Thousand Pounds for the London Hospitals (W.), 56
Sleep and its Disorders (C.), 147, 265
Small-pox (P.), 202
Small-pox Hospital a Nuisance? Is a, 175
Snake Poisons (P.), 183
Snare (Rhinology), A New (P.), 83
Social Problems, 4, 42, 58, 94
Sociology, Modern, 94, 144, 212, 245, 262, 294, 310, 376, 410
Soda, Bicarbonate of (in Gastric Affections) (P.), 9
Sodium Benzoate (in Laryngitis) (P.), 65
Spina Bifida (P.), 269

Spinal Abscess Chronic (P.). 269
Spinal Cord, The Localisation of Tumours of the (C.). 313
Spine, Surgery of the (P.). 269
Splanchnic Nerves (P.), 415
Spleen, The Treatment of Ruptured Spleen, 350
Spurious Hospitals, 59
Squint, 3, 41 93, 161, 245, 261
Squint, Latent, 341, 375
Statistics, The Fallacies of Crude. 22
Sterilised Milk, Penny Bottles of, 25
Stock Exchange and the Hospitals, The, 71
Stomach, Dilatation of the (P.), 9
Stomach, Diseases of the (P ), 8, 28, 382
Stomach, Hour-Glass Contraction of the (P.), 399
Story of the Insane from Year to Year (W.), *see Insane*
Strasburg Hospital for Women (W.), 171
Student of Medicine, The (*Appointments, Vacancies, Notes, &c.*). 19, 37, 53. 71, 89, 108, 140. 157, 174, 191, 208, 224, 242, 257, 273, 289, 306, 322, 338, 354. 372. 388, 405, 422, 439
Study of Man, The, 127, 195
Study of Man at the British Association, The, 23
Stuffy Railway Carriages, 263
Stypticen, A New Hæmostatic (P.), 334
Subphrenic Abscess (P.), 252
Sudden Death, On, 295
Sugars, The Digestion of (P.), 167
Suicides, Increase of English. 343
Sunderland Infirmary (A. H.), 36
Sunshine, Air and Germs, 359
Sunshine on the South Coast, 356
Sunstroke, 5
Supra-Renal Bodies (P.), 363
Surgery, Abdominal (P.), 321
Surgery, Brain (P.). 10
Surgery, Cerebral (P.), 300, 316
Surgery of the Chest (P.), 251
Surgery, General (P.), 150, 161, 183
Surgery, Genito-Urinary (P.). 116, 132, 185
Surgery of the Liver and Gall-Bladder (P.), 29, 417
Surgery of the Nerves (P.), 11, 234
Surgery of the Œsophagus and Stomach, 398
Surgery, Orthopædic (P.), 332
Surgery, Rectal (P.), 268
Surgery of the Spine (P.), 269
Surgery of the Ureter (P.), 118
Surgery of the Vermiform Appendix (P.), 431
Surgical Fees at Charity Hospitals, 260
Symphysiotomy (P.), 102
Syphilis (P.), 186
Tees, Floating Hospital on the (P.), 255
Testes, Strangulation of the (P.), 185
Tetanus (P.), 299
Therapeutic Notes (P.), 13, 334, 349, 384, 400
Thrombosis, Primary, of Superior Longitudinal Sinus (P.), 301
Thymus Gland and Grave' Disease, The (P.), 64
Thyreo-Antitoxin (P.), 349
Thyroid Gland (P.), 49
Tight Lacing, Some Effects of (C.), 165
Tilbury, A New Cottage Hospital at (W.), 239
Tinea (P.), 433
Tonsil, Giant Cell Systems in the (P.), 12
Tonsil, Lingual (P.), 31
Tonsilitis, Chronic, of Coli Bacillary Origin (P.), 31
Tonsils, Latent Tuberculosis of the Three (P.), 12
Tonsils, New Scissors for Excising (P.), 31
Tonsils, Role of the, in Infection by Pyogenic Organisms (P.), 12
Topographical Anatomy (P.), 331
Torticollis, Spasmodic (P.), 11
Trade Unionism in Medicine, 295
Traumatic Neurasthenia, 427
Traumatic Neuritis (P.), 235
Treves' Method (Pott's Disease), (C.), 97
Trigeminal Neuralgia, Operations for (P.), 235
Trigeminus, Neurectomy of the (P.), 11
Tubercular Meningitis (P.), 317
Tuberculosis (P.), 217, 253
Tuberculosis in Cattle, 126
Tuberculosis, Laryngeal, The Surgical Treatment of (P.), 253
Tuberculosis, More Dangers from, 311
Tumours (P.), 433
Tumours, Cerebral (P.), 317
Tumours, Disappearing Abdominal (P.), 399
Tumours of the Breast (P.), 203
Tumours of the Spinal Cord, The Localisation of (C.), 313
Typhoid (P.), 148, 299; (C.), 216
Typhoid and Tenancy, Another Legal Decision, 395
Typhoid, Laryngeal Lesions of (P). 65
Ulcer, Gastric, Perforation of a (P.), 399
United States Army Medical Department (W.), 336
Unproved Remedies, Restrictions in the Use of, 141
Ureter, Surgery of the (P.), 118
Uterus, Rupture of the (P.), 102
Vaccination, Leg *versus* Arm (C.), 379
Vaccination Question, The, 390
Vaccination, The Case for (C.), 181
Vaccines, The Multiplication of, 210
Valparaiso, New Hospital for (W.), 221
Vertebral Canal, Tapping the (P.), 10, 269
Vermiform Appendix, Surgery of the (P.), 431
Vertigo, A New Form of Rotatory (P.), 134
Vitalism, The Old and the New, 408
Vocal Cord, Angio-keratoma of the Right Vocal Cord (P.), 50
Vocal Cord, Structure of so-called Fibroma of the (P.), 65
Vocal Cords, Abductor Paralysis of the (P.), 50
Waldo (Dr.) and the Salvation Army, 142
Walton on-Naze, A New Broom at, 129
Water, The Chemical Sterilisation of, 145
Water, The Vindication of, 162
Water-drinker Three Centuries Ago, The, 128
Whitechapel Guardians and Local Doctors, The, 279
Whitehead's Operation (P.), 268
Who is to Judge? 327
Why do not Women Insure their Lives? 327
Wigan Royal Albert Infirmary (A. H.), 36, *and* (W.), 436
Winning Races, The Evolution of the, 279
Winter Quarters, 110
Wood Green Cottage Hospital (W.), 104

### THE SPECIAL SUPPLEMENT FOR NURSES.

American Letter, Our, xxiii, li, c, cxxxiii, clx. clxxi., ccxxiv
Appointments, xviii, xxxii, xlii, liii. lvii, lxxi lxxxi, xc, xcvii, cxvi, cxxiv, cxxxvi, cliv. clxii, clxxii, clxxxv, cc, ccix, ccxxvi, ccxxxv
Appointments, Minor, xv, xxiii. xxx, xxxv, xxxvi, xlix, lx, cii, cviii, cxxxvi, cxlii, clxx. cxciii, ccxxvi, ccxxxiv
Asylum Lectures, lxxxi
Barnardo's (Dr.) Entertainment at the Albert Hall, cxxxv
Bazaar at Devonport, clxxxii
Bedford College for Women, London, xvi
Book and its Story, A, clxxiv, clxxxiii, cciv, ccxxxiii
Book World for Women and Nurses, liv, cxxvi clxvi, clxxxvi, cxcvi, ccxvi, ccxxxvi
Books for the Young, xcii
Bournville, cciv
Burdett's Official Nursing Directory, ccxxix
Canine Collectors for Hospital Funds, xxviii
Chicago Woman's Club, clxxi
Christmas: What Christmas Means to Hospital Officials, lxxxvi
Christmas and New Year Gifts, xc
Christmas Books, xcii
Christmas Entertainments, cxiv, cxxii, cxliii
Christmas in Hospital, xcix
Christmas in the Provinces, cxv
Coincidence, A, liv
County Nursing Association, lxxxv
Death in Our Ranks, cxix. ccv
Difficulties of the R.B.N.A. *See p* clxi
District Nurse at Cockermouth, c
District Nursing in Ireland, ccxxv
Doctors and Nurses at Cork, ccxiv
Doll Show and Competition, clxxii
Dress and Uniforms, v, xlv, cxxi, clxxxi, ccxxxi
Dundee Royal Lunatic Asylum, lxxxii., lxxxviii
East End Mothers' Home, ccxxv
East London Nursing Society, ccxi
Entertainments, cxxxiv, cl
Everybody's Opinion, xii, xvii, xxiv, xxxi. xxxvii, xlvi, lii, lxi, lxxx, xciv, ci, cviii, cxxv, cxxxv, cxlv, clv, clxv, clxxv, clxxxv, cxcv, ccv, ccxv, ccxxv, ccxxxiv.
First Aid at Aldershot cviii, cxii
Florence Nightingale (*Poem*), xvi
Food: With Special Relation to the Sick, cvi
Food, Talks About, cxciv, ccii
French Schools for Trained Nurses: Their Origin and Organisation, vii, lxxii, cxvi
Funeral of an Army Nurse with Military Honours, cxliv
Glasgow Nurses' Co-operation, lxxxviii
Help the Nurses to Help the Sick, lxxxix
Holidays and Health, viii
"Home for the Dying," A New, clxxxii
Homeward Bound, cc
Hospital, A Swiss, clxxxiv.
"Hospital, The" Convalescent Fund, xvi
How the Public can Protect Itself. lxxxii
Lady Cyclists "on a Firm Social Basis," clx
Lecturer on Sanitation, Some Experiences of a, cxcii
Lectures on Nursing, xcvii, cv, cxi, cxix, cxxix, cxxxix, cxlix, clix, clxix, clxxix, clxxxix, cxcix
Lectures to Nurses, cxxxv
London Nurses' Training Schools. xxix, l
Matrons and "Private Practice" (!), cxliv
Medical Study for Women in Russia, The, lxxxi
Medical Women, A Tribute to, cviii
Medico-Psychological Association, cxxii
Melbourne, News from, ccxxxv; Notes from, xi
Midwife and the Midwifery Nurse, The, cxli
Midwifery Papers, ccix
National Hospital for Paralysis and its Lady Superintendent, The, ccxx
Needlework Competition, Our, cvii
Neuralgia and Its Treatment, iv, xxii
New South Wales, Training in, ccxxxii
News from the Nursing World, i, xiii, xix, xxv, xxxiii, xxxix, xlvii, lv, lxix, lxxv, lxxxiii, xcv, ciii, cix, cxvii, cxxvii, cxxxvii, cxlvii, clvii, clxvii, clxxvii, clxxxvii, cxcvii, ccvii, ccxvii, ccxxvii
Nightingale, Miss, in Buckinghamshire, lxxxvii
Notes and Queries, iv, xvi, xxi, xxxii, xliii, liii, lviii, lxxiv, lxxxi, xciii, cii, cviii, cxv, cxxv, cxxxvi, cxlvi, clv, clxv, clxxv, clxxxvi, cxcvi, ccvi, ccxv, ccxxvi, ccxxxv
Notification and Registration of Sickness, A National Scheme for the xcviii
Novelties for Nurses. vi, xxii, xxxvii, liv, lxii, lxxiv, cvii, cxvi, cxl, clxiv, clxxxv, cxcii, ccxiv, ccxxxv
Nurse, The Half-trained, lviii
Nurse, The Trained—An American View, ccxxiii
Nurses, The Comforts of, lxxviii
Nurses, The Position of, clxxxv
Nurses and Their Competitors, cxx
Nurses' Co-operation, The, clx, clxx
Nurses in the Provinces, ix
Nurse's Life, Practical Aspects of a, cxiii
Nurse's Uniform, The Misuse of a, ccxxxiv
Nursing: Its Ideals and Realities (*Poem*), clxiii
"Nursing Notes" Exhibition, xlix
Nursing, Private, cxliii, clxxx, ccxii, ccxxix
Nursing Question as seen in England and Germany, Certain Aspects of the, clxxiii, cxcii, cciii, ccxxx
Nursing in Canada, clxxi
Nursing in Florence, li, lxxix, cl
Nursing in India, xliv
Nursing in Ireland, iii
Nursing in Rome, vi, xxvii
Nursing at the Spalding Union Infirmary, lx
Ockley Scheme, The, x
Oriolet Hospital, The, cliv
Ormskirk, A Sad Occurrence at, xliv
Pasteur Institute, A Visit to the, xvii
Penrith Fever Hospital, The, lx
Pension Fund. *See Royal National*
Pension Fund, Twenty-three Thousand Pounds for the, xxxvi
Physiology for Nurses, Elementary, iii, xv, xxi, xxvii, xxxv, xli, xlix, lvii, lxxi, lxxvii, lxxxiv
Pirated Advertisements, cxc
Practical Points, clxxxiv, cxcv, ccxxv, ccxxxv
Presentations, liii, lix, lxxxvii, cxv, cxxiv, cxxxiii, cxl, cxc, ccxvi
Princess Maud Marriage Present Fund, The, liii, lxiii, lxxix, lxxxviii, cxvi
Princess Maud, Nurses' Gift to the, cxci, cc, ccix
Private Hospital in Australia, A, x
Probationers at York County Asylum, lxxxv
Queen Victoria Jubilee Institute for Nurses, cxxxiii
Reading to the Sick. For, xxxviii, lxii, lxxxii, cxxvi, cxxxvi, cxlvi, clvi, clxvi, clxxvi, clxxxvi, cxcvi, ccxvi, ccxxvi, ccxxxvi
Registration of Nurses, cxxv, cxxxi
Riviera Nursing Home, A, clxxi
Royal British Nurses' Association, xvi, xxx, xxxii, facing p. xxxvii; xli, liii, lxxi, lxxix, xci, cxxiv, cxxx, cxxxvi, cli, clxi, cc, ccix, ccxxiv, ccxxx
Royal London Ophthalmic Hospital, Moorfields, cxxx, cxliv
Royal National Pension Fund for Nurses, xliii, lxxiii, cxci, cc, ccx, ccxxii
St. Catherine's Home, Bradford. cviii
St. George's Hospital, xcviii
St. John Ambulance Association Nursing Corps, clxx
St. Luke's Home, Vancouver, clxxxii
St. Thomas's Hospital, Opening of Wards at cxci
Sleep and Sleeplessness, cxxiv
Superintendents of Training Schools' Association, cci
Temperance for Nurses, clxxxii
Thermometer, The Evolution of the, cxl
Tommy Atkins, One Aspect of, xi
Training at Chelsea Hospital for Women, lx
Training School Registries, ccxiii, ccxxi
"Truth" Doll and Toy Show, cvi
Uniform, A Suggestion for, cii
Urine Testing, On, xxviii, xlii, l
Wants and Workers. xxxii, xxxv, l, lx, lxxvii, cxviii, cxxiv, cliv, clxiv, clxxiii, clxxxii, cxcv, ccxiii, ccxx, ccxxxiv
Where to Go, viii, xxiii, xxx. xxxvi, xli, li, lvii, lxxiv, lxxvii, lxxxvii, cviii, cxxvi, cxlii, clvi, clxi, clxxvi, clxxxiv, cxcv, ccvi
Winter Exhibitions, cxiii, cxxxiv
Women Workers in New England Hospitals, clxxii
Workhouse Infirmary and Hospital Visiting, clxii
Workhouse Infirmary Nursing, cxc
Working Women, The Present Position of, clxiv

Printed by W. H. and L. Collingridge, "City Press," 148 and 149, Aldersgate Street, London, E.C.

**Notices of Births, Deaths, and Marriages** are inserted in this column at a charge of 2s. 6d. for an announcement not exceeding four lines.

**Notice to Advertisers and Subscribers.**

All Advertisements, Orders for Copies of The Hospital and Business Communications should be addressed to **The Manager, NOT TO THE EDITOR**, The Hospital, 428, Strand, London, W.C.

The Cost of Subscription, post free, is as follows :—Per week, 2½d.; per quarter, 2s. 8d.; per half-year, 5s. 3d.; per annum, 10s. 6d. Foreign:—Per Annum, 15s. 2d.; payable, in all cases, in advance.

**NOTICE TO CORRESPONDENTS.**

All MS., Letters, Books for Review, and other matters intended for the Editor should be addressed The Editor, The Lodge, Porchester Square, London, W.

The Editor cannot undertake to return rejected MS., even when accompanied by stamped directed envelope.

**"Burdett's Hospital Annual," 1895,**

Sixth year of publication. 950 pp. Scarlet cloth, gilt lettered. Price 5s. A most complete guide to British, Colonial, Indian, and American Hospitals, Dispensaries, Nursing and Convalescent Institutions, and Asylums. A few copies of the "Annual" for 1894 may still be obtained on application to the Publishers, The Scientific Press (Limited), 428, Strand, London, W.C.

**NOTICE.—Vol. XVIII. of "The Hospital" (April to September, 1895), in handsome cloth binding, will be on sale at the Office of this Journal next week. Price 6s. Cases for binding the Numbers contained in this Volume, 1s. 6d. Index to Vol. XVIII., 2½d., post free. The Monthly Part for October is now ready, price 6d.; by post, 8d.**

### Notes and News.

### THE INTRODUCTORY ADDRESSES AT THE MEDICAL SCHOOLS.

### St. George's Hospital.

### Middlesex Hospital.

### Scraps and Gleanings.

It has been suggested that there is great need of a cottage hospital at St. Anstell, in the Truro district.

The Finance Committee of the Hertford and Ware Joint Hospital Board record a balance with the treasurer of over £300.

Queen Amelie of Portugal is now studying medicine, and goes into the intricacies of the science with the zeal of a professional man.

The church parade of the Leominster friendly societies took place this month. There were 600 processionists. Over £24 were collected *en route*.

At the meeting of the Committee of Management of the Royal Hospital for Incurables, at Dublin, it was shown that the hospital is in the debt of the bank to an extent of over £1,000.

The Wrexham Hospital Saturday appeal met with a successful issue, over £90 on behalf of the Wrexham Infirmary being subscribed one day by aid of collecting-boxes and other agencies.

The Sunderland Infirmary continues to do important work. The number of patients have increased during the year, so that the benefits of the institution in that respect had been very materially extended.

A handsome legacy has been left to the Académie de Médicine by M. Charles Louis Herpin with the object of founding a prize annually for the best work on nervous diseases, to be called the "Herpin (of Geneva) Prize."

A cheque of £21 has been sent to St. Mark's Ophthalmic Hospital and Dispensary, at Dublin, from Mr. Nicholas Lynch. The governors of this institution draw attention to the great increase in the work done by the hospital.

The annual meeting of subscribers of the Devizes Cottage Hospital and Provident Dispensary has just been held. In the report we notice that the statement of accounts is not quite so flourishing as it has been of late years.

A movement has been started by a number of influential residents in Hampstead to secure the estate of the late General Fraser as a park and recreation ground. The proposed park is situated by Finchley Road and has an area of 13 acres.

It is gratifying to notice that at Macclesfield the various local schools are accepting the suggestions of the governors for the placing of subscription boxes in their rooms for the benefit of the children's ward of the General Infirmary.

One of the laws in Florida regarding contagious diseases provides that any person maliciously giving false reports about the existence of any contagious disease shall be punished by a heavy fine, or be imprisoned in the county jail for not less than three months.

The Hospital Saturday collection at Folkestone was of a satisfactory nature this year, £314 having been collected, as against £273 in 1894. We are glad to state this, as erroneous information as to figures had previously reached us, of a character not according to the actual facts of the case.

A special appeal is being issued by the treasurer of the Victoria Cottage Hospital, Guernsey, in aid of the funds of this deserving little institution. A manful struggle is made by the authorities to keep the expenditure within the income; last year closed with a balance in favour of, instead of against, the hospital.

It is gratifying to the board of the Sheffield General Infirmary to record the result of the 19th Hospital Saturday as benefiting the charity to the extent of £650. The continued interest taken by the industrial classes in the institution is worthy of all interest.

A meeting has just been held at Hungerford for the purpose of receiving a statement of receipts and disbursements in connection with the late efforts made in the town to raise money for the hospitals. It was found that after all expenses had been paid there would be a balance for disposal of £72.

The directors of the Royal Infirmary at Aberdeen have come to an end of their difficulties about procuring a suitable site for the erection of their proposed New Convalescent Home. Four acres of ground have been secured at Hillhead, a site near the high service reservoir, and arrangements are being made already for obtaining water from this city supply.

In connection with the proposed erection of a new infectious diseases hospital for Edinburgh, we understand that Bailie Pollard, convener of the Public Health Commitsee, has just provisionally purchased on behalf of Edinburgh Corporation, the farm of Colinton Mains. The sum to be paid, if the Council promulgate the convener's action, will be £20,000.

A French paper gives the leech as an example of a good barometer. A leech, placed in a bottle of water, lies rolled up together at the bottom of the bottle when it is going to be "fair" weather, coming to the surface if it is going to be variable, rushing rapidly about the bottle in the coming of wind; in the case of a coming storm it is said to roll over rapidly.

An interesting presentation was made to Mr. Studt, of Swansea, lately, by the Leominster Cottage Hospital Fund Committee, at the Corn Exchange, Leominster. Mr. Studt has been a beneficent patron to the hospital, and his generous services in giving fêtes in its favour have been highly appreciated. The presentation made to him on the occasion referred to above took the form of a richly-illuminated address.

An excellent law has lately been passed by the Legislature of the State of New York, providing that all hospital buildings which are used for general hospital purposes, or asylums, or any buildings of this nature that are more than two storeys high, shall have properly constructed iron stairways on the outside of the walls, with doorways leading on to them. These provisions are wisely advanced in the event of fires breaking out.

There is quite an agitation at Rhyl on account of the projected erection of the Alexandra Hospital. A meeting of protesting ratepayers and property owners met last week, where it was moved, "That this meeting has heard with great regret and concern of the proposal to erect a new hospital building of enormously increased dimensions on the present site, and desires now to express its emphatic opinion that if such a proposal is carried out it will be highly detrimental to the future welfare of the town."

### The Book World of Medicine and Science.

The Twentieth Century Practice of Medicine. (London: Sampson Low, Marston, and Co.)

### The Student of Medicine.

### APPOINTMENTS.

Dr. Bateman, appointed Medical Officer for the Workhouse and the Walsingham District of the Walsingham Union. F. Dendle, M.B., C.M.Edin., appointed Medical Officer for the Glencraig Colliery. M. J. Doidge, B.A.Camb., M.R.C.S.Eng., appointed Medical Officer for the No. 3 District of the Wells Union. W. Foster, B.A., M.B., D.P.H.Camb., M.R.C.S.Eng., appointed Medical Officer of Health for the Shipley Urban District. J. A. Fox, L.R.C.P.Lond., L.R.C.P.Edin., L.R.C.S.Edin., L.F.P.S.Glasg., L.S.A.Lond., appointed Medical Officer and Public Vaccinator for the No. 1 District and Workhouse, Penzance Union. J. C. Garman, L.R.C.P., L.R.C.S., appointed Medical Officer and Public Vaccinator for the No. 3 (Warbleton) District of the Hailsham Union. D. W. Girvan, M.B., C.M.Glasg., appointed Medical Officer for the Cardiff South District of the Cardiff Union. W. Gordon, M.B., appointed House-Surgeon to the Salisbury Infirmary. E. H. Helby, M.R.C.S., L.R.C.P., D.P.H., appointed Resident Medical Officer to the Croydon Borough

### THE STUDENT OF MEDICINE—*continued.*

Hospital. J. Macdonald, M.A., M.B., C.M.Edin., appointed Medical Officer of Health for the Carlisle Rural District. H. Macintyre, M.B., C.M.Glasg., appointed Senior Assistant Medical Officer to the Shoreditch Infirmary. A. H. Mason, L.R.C.P.Lond., M.R.C.S.Eng., appointed Medical Officer for the Walton and Oatlands District of the Chertsey Union. Dr. Matthews, appointed Medical Officer for the Sixth District of the Mansfield Union. J. W. Pare, M.D., C.M.Edin., L.D.S., appointed Lecturer on Dental Anatomy and Physiology to the National Dental Hospital. Dr. Sharp, appointed Medical Officer for North Marine. A. Walker, M.B., C.M.Glas., appointed Medical Officer of Health for the Weetslade Urban District. F. Ward, M.B., C.M.Edin., appointed Second House Surgeon to the East Suffolk Hospital, Ipswich. R. Yelf, M.B., C.M.Edin., appointed Medical Officer and Public Vaccinator for the Moreton District of the Shipston-on-Stour Union.

### VACANCIES.

The following vacancies are announced this week, the amount of salary in each case being given after the name of the institution:—

#### Resident Medical Officers.

Essex and Colchester Hospital.—House Surgeon, doubly qualified. Salary £80 per annum, with board and lodging in the hospital. Applications to the Committee by October 18th.

Hospital for Consumption and Diseases of the Chest, Brompton.—Resident House Physicians. Applications and testimonials to W. Theobald, Secretary, by October 10th.

Royal Alexandra Hospital for Sick Children, Dyke Road, Brighton.—House Surgeon; doubly qualified. Salary £80 per annum, with board, lodging, and washing; no stimulants. Applications to the Chairman of the Medical Committee by October 6th.

Royal Free Hospital, Gray's Inn Road, W.C.—Resident Medical Officer (House Physician), doubly qualified. Appointment for six months, but eligible for re-election. Board, residence, and washing provided. No salary. Applications to the Secretary by October 19th.

South Devon and East Cornwall Hospital, Plymouth.—House Surgeon. Salary £100, with board and residence. Applications and testimonials to J. Walter Wilson, Honorary Secretary, by October 12th.

#### Non-Resident Medical Officers.

Carnarvon Joint Sanitary District.—Medical Officer of Health, must be between 25 and 40 years of age, doubly qualified. Must devote his whole time to the duties, and have a knowledge of the Welsh language. Appointment for five years. Salary £694 per annum, inclusive of all expenses, except those incurred for such books, stationery, and apparatus required in the performance of the duties. Applications, endorsed "Application for Office of M. O. Health," to J. H. Thomas, Clerk to the Joint Committee, 14, Market Street, Carnarvon, by October 16th.

East London Hospital for Children and Dispensary for Women, Glamis Road, Shadwell, E.—Assistant Physician to see out-patients. Must be a Fellow or Member of the Royal College of Physicians of London. Applications to Thomas Hayes, Secretary, by October 26th.

Glasgow Maternity Hospital.—Obstetric Physician and Assistant Obstetric Physician. Applications to Arthur Forbes, Secretary, 146, Buchanan Street, Glasgow, by November 8th.

London Hospital, Whitechapel, E.—Medical Electrician; must be qualified and registered under the Medical Act. Applications to G. Q. Roberts, House Governor, by October 19th.

Metropolitan Hospital, Kinsland Road, N.E.—Assistant Surgeon. Must be a Fellow of the Royal College of Surgeons of England. Applications and testimonials to Charles H. Byers, Secretary, by October 7th.

Parish of St. Mary, Islington.—Resident Assistant Medical Officer for the Workhouse and Infirmary. Salary £80 per annum, with rations, apartments, and washing, or an allowance in lieu or same. Applications to Edwin Davey, Clerk, by October 8th.

**On Modern Progress in Ophthalmic Medicine and Surgery.** By R. Brudenell Carter, F.R.C.S. - - - 3

**Medical Progress and Hospital Clinics—**

The Treatment of Patients after Abdominal Operations - - 7

Progress in Medicine.—Diseases of the Stomach - - 8

Progress in Surgery.—Brain Surgery - - - - 10

Surgery of the Nerves - - - - - - - - 11

Progress in Laryngology.—Pharynx - - - - - 12

Therapeutic Notes.—Argonine and Argentamine - - 13

New Appliances and Things Medical - - - - - 14

**Social Problems.**—The Criminal Type - - - - - - 4

**Annotations.**—The Classification of Paupers—Sunstroke—The Death of M. Pasteur - - - - - - - - - - - 5

**Notes and News** - - - - - - - - - - - - - 6

**The Institutional Workshop—**

Hospital Expenditure.—The Commissariat.—I. - - - 15

Practical Departments - - - - - - - - - 16

Hospital Festivals, Meetings, &c. - - - - - - 17

Construction Notes - - - - - - - - - - 17

**The Editor's Letter Box** - - - - - - - - - 18

**Scraps and Gleanings** - - - - - - - - - 18

**The Book World of Medicine and Science** - - - - 19

**Nursing in Ireland** - - - - - - - - - - - iii

**Neuralgia and its Treatment.**—I. - - - - - - - iv

**Dress and Uniforms.**—St. Mary's Hospital - - - - - v

**Novelties for Nurses—Nursing in Rome.**—III. - - - vi

**French Schools for Trained Nurses: Their Origin and Organisation** - - - - - - - - - - - - vii

**Holidays and Health.**—Ireland—**Where to Go** - - - - viii

**Nurses in the Provinces.**—Training - - - - - - ix

**The Ockley Scheme.**—The Name of Nurse - - - - - x

**One Aspect of Tommy Atkins—Notes from Melbourne** vi

**Everybody's Opinion** - - - - - - - - - - - xii

Now Ready, post 8vo., 8s. 6d., post free, plentifully Illustrated with Coloured Plates, and in Black and White.

### DISEASES of the Upper Respiratory Tract, THE NOSE, PHARYNX, and LARYNX.

By **P. WATSON WILLIAMS, M.D.Lond.**, Phys. in Charge of Throat Dept., Bristol Royal Infirmary.

"This work is certain to receive the recommendation of all teachers. Deserving exceptional praise is the beauty and typical character of the black and white drawings."—*British Medical Journal.*

"Clearly written, concise, complete, well illustrated. . . We strongly recommend the book as a practical guide to diseases of the nose and throat."—*Lancet.*

"We do not remember having read any book on a speciality in medicine that has given us such sincere pleasure."—*Clinical Journal.*

"A complete picture of our knowledge of the region, based upon the most recent scientific work."—*Internat. Cent. f. Laryng. u. rhin.*

Bristol—J. WRIGHT and CO. London—SIMPKIN, MARSHALL, and CO. (Limited).

Edinburgh: Oliver and Boyd.
London: Simpkin, Marshall, and Co. (Limited).

Recently Published, 8vo., with Illustrations, price 18s.

**THE DISORDERS OF SPEECH.** By John Wyllie, M.D., F.R.C.P.Ed., Physician to the Royal Infirmary, Edinburgh.

"A book which for comprehensiveness, clearness of style, and practical usefulness is certain to hold its own for a long time as a standard authority. . . . The chapter on stammering is the best and most original in the book. . . . There is throughout evidence of careful and mature thought."—*British Medical Journal.*

"We may say that for fulness of detail and clearness of description these chapters [on Aphasia] have few, if any, equals in our language."—*Dublin Journal of Medical Science.*

8vo., price 7s. 6d., with Illustrations.

**THE NEUROSES OF DEVELOPMENT.** By T. S. Clouston, M.D., F.R.C.P.E., Physician-Superintendent, Royal Edinburgh Asylum for the Insane.

"It is a work which is full of interest, and is likely to have a very wide sphere of influence, and we are sure that no one can look into it without having suggested to him some fresh point from which to regard many of the commonest nervous disorders."—*Lancet.*

### WORKS PUBLISHED BY JOHN WRIGHT AND CO., BRISTOL.

Third Year. Now Ready. 5s. 6d., post free. In flexible leather, 2 Pockets and Fastener; gilt edges. Copies on approval to Medical men.

**Wright's Improved Visiting List, 1896.** For Physicians, Surgeons, and Consultants. Requires writing up only once a month.

(Design registered). "About the most convenient and complete list with which we are acquainted."—*Liv. Med. Jour.*

**Wright's Improved Perpetual List.** The same form and price as above, but may be commenced at any time. This is issued now for the first time by request.

Now Ready. 8vo., cloth, bevelled boards. 10s. 6d. net, post free.

**Leprosy: In its Clinical and Pathological Aspects.** Illustrated with 13 Plates—Platinotypes and Chromo-lithographs. By Dr. G. ARMAUER HANSEN, Inspector-Gen. of Leprosy in Norway, and Dr. CARL LOOFT, formerly Assist.-Physician to the Lungegaard's Hosp. Translated by NORMAN WALKER, M.D., F.R.C.P. Edin., Assist.-Phys. for Dermatology, Edinb Roy. Infirm.

"I have said enough to show that the work is indispensable to every author who deals with Leprosy, and when I add that the letterpress fills only 125 short pages, and that in these pages every important problem connected with Leprosy is duly weighed, I need hardly add that it should be read by every physician who is interested either in pathology or dermatology."—Geo. Thin, M.D., in *Brit. Journ. of Dermatology.*

"A very important contribution to the subject with which it deals."—*Times.*

"Contains much valuable information about this terrible disease."—*Lancet.*

"Few men are better fitted than the discoverer of its bacillus to enlighten us on the course of Leprosy, and very few have taken such full advantage of their opportunities of studying it."—*Edin. Med. Journ.*

Post 8vo. Illustrated with Coloured Plates and in Black and White. 8s. 6d., post free.

**Diseases of the Nose, Pharynx, and Larynx.** By P. WATSON WILLIAM M.D.

"This work is certain to receive the recommendations of all teachers. The student or practitioner will have difficulty in finding elsewhere so much instruction conveyed readably in so little space."—*Brit. Med. Journ.*

Second Edition. 8vo., cloth. Illustrated. 4s. net, post free.

**Urinary Surgery for Students and Practitioners.** By E. HURRY FENWICK, F.R.C.S.

Being No 1 of Wright's "Epitomes of Modern Progress in Surgery."

"Those who wish for trustworthy information will find what they require."—*Brit. Med. Journ.* "Of real practical value."—*Brit. Med. Journ.*

Second Edition. Revised and enlarged, cloth, 8vo. Price 4s. 6d., post free.

**Ophthalmological Prisms and the Decentering of Lenses.**

A Practical Guide to the Uses, Numeration, and Construction of Prisms and Prismatic Combinations, and the Centering of Spectacle Lenses. Illustrated by 69 Original Diagrams. By ERNEST E. MADDOX, M.D.

"The writer has a happy faculty of explaining difficult subjects in a clear and concise manner, and throughout the book all difficult mathematical problems have been avoided."—*Brit. Med. Journ.*

Fourth Edition. Revised. Waistcoat Pocket Size. Paper covers, 1s., post free.

**Golden Rules of Surgical Practice.** For the use of Dressers and Junior House Surgeons. By E. HURRY FENWICK, F.R.C.S.

"Sound, short, and serviceable."—*Lancet.*

Third Edition. 10s. 6d., post free. With 235 Engravings.

**Pye's Surgical Handicraft.** A Manual of Minor Surgery for the use of General Practitioners, House Surgeons, Students, and Surgical Dressers. By WALTER PYE, F.R.C.S. Revised and Edited by T. H. R. CROWLE, F.R.C.S. With Special Chapters on Aural Surgery, Teeth Extraction, Anæsthetics, &c. By Messrs. FIELD, HOWARD HAYWARD, and MILLS.

Seventh Edition. 2s., post free. Pocket Size. 80 Illustrations. Adopted by the St. John Ambulance Association.

**Elementary Bandaging and Surgical Dressing.** With Directions concerning the immediate Treatment of Cases of Emergency. Mostly condensed from Pye's "Surgical Handicraft." By WALTER PYE, F.R.C.S.

Large 8vo. Reprint. Cloth boards. Illustrated. 6s., post free.

**The Practice of Hypnotic Suggestion.** An Elementary Handbook for the use of the Medical Profession. By GEORGE C. KINGSBURY, M.A., M.D. (University of Dublin).

Small 4to. Cloth lettered. Price 2s. 6d., post free.

**Rheumatism: Some Investigations respecting its Cause,** Treatment, and Cure. By PERCY WILDE, M.D.

Pocket size, limp covers, 2s., post free. With numerous Illustrations, reduced from the larger work by the same Author.

**Stretch Dowse's Primer of the Art of Massage (for learners).** By THOMAS STRETCH DOWSE, M.D.

Just Published, with numerous Illustrations, 8vo., cloth, 2s. 6d., post free.

**Eye-Sight and School Life.** By SIMEON SNELL, F.R.C.S. Edin., Ophthalmic Surgeon General Infirmary; Lecturer on Diseases of Eye at School of Medicine; and Ophthalmic Surgeon to School for Blind, Sheffield; Consulting Ophthalmic Surgeon to Rotherham Hospital.

Now Ready. 8vo., cloth. 3s. 6d., post free.

**Pulmonary Consumption: Its Nature and Absolute Cure** by Dry-Air Inhalation. A Practical Treatise dealing with the Cause of Consumption, showing How it can be Prevented and Successfully Treated. By JOHN J. HARTNETT, M.D.

Third Edition, 8vo., thick paper covers, 1s. 6d.; or cloth, 2s. 6d., post free. May be safely recommended.

**Our Baby: A Book for Mothers and Nurses.** By Mrs. LANGTON HEWER, Diplomée Obstetrical Society, London, late Hospital Sister. Author of "Antiseptic Nursing."

Now ready, long 8vo., stiff covers, flush, 1s. 6d., post free.

**Maladies of Old Age and their Treatment.** By G. H. DOUDNEY, M.B., M.R.C.S.Eng. Author of "The Water-Cure in the Bedroom, or Hydropathy at Home."

One Hundredth Thousand. Samples and Price List on application. 50, 5s; 100, 8s. 6d.; 500, 13s.; 1,000, 24s.

**Wright's Registered Pocket Charts, for Bedside Case Taking.** Folds into the Visiting List. Compiled by ROBERT SIMPSON, L.R.C.P., L.R.C.S.

Samples and Prices on application. The cheapest issued.

**Registered Combination Temperature and Diet Charts.** With Clinical Diagrams specially arranged for Hospital use. Designed by ROBERT SIMPSON, L.R.C.P., L.R.C.S.

**Wright's Medical Account Books.** Samples and Prices on application.

1s. each, 10s. per doz., post free.

**Wright's New Prescription Books.** Single or Duplicate. Gold stamped, round corners, gilt edges, printed and perforated. The neatest books published. Full Prices on application.

Bristol: JOHN WRIGHT & CO. London: SIMPKIN, MARSHALL, HAMILTON, KENT, & CO. (Limited).

### MR. BURDETT'S BOOKS ON HOSPITALS.

"Mr. Burdett's monumental work on Hospitals."—*Times*.

In Four Volumes, with a Portfolio of Plans, cloth extra, bevelled. Royal 8vo. Top gilt. Price £8 8s. complete (see below).

### HOSPITALS AND ASYLUMS OF THE WORLD: Their

Origin, History, Construction, Administration, Management, and Legislation; with Plans of the chief Medical Institutions accurately drawn to a uniform scale, in addition to those of all the Hospitals of London in the Jubilee year of Queen Victoria's reign. By HENRY C. BURDETT, formerly Secretary and General Superintendent of the Queen's Hospital, Birmingham; and the "Dreadnought" Seamen's Hospital, Greenwich; Founder of the Home Hospitals Association for Paying Patients, the Hospitals Association, and the Royal National Pension Fund for Nurses; Author of "Pay Hospitals of the World," "Hospitals and the State," "Cottage Hospitals," &c., &c.

*In four volumes, and a separate Portfolio containing some hundreds of Plans.*

| | |
|---|---|
| Price of the Book complete - - - - - - - - - | £8 8 0 |
| Vols. I. and II. (only).—Asylums and Asylum Construction | 4 10 0 |
| Vols. III. and IV.—Hospitals and Hospital Construction —with Portfolio of Plans - - - - - - - - - | 6 0 0 |
| Portfolio of Plans separately- - - - - - - - - | 3 3 0 |

Sixth year of Publication. 950 pp., Crown 8vo. Red Cloth Gilt. Price 5s.

### BURDETT'S HOSPITAL AND CHARITIES ANNUAL, 1895,

Being the Year Book of Philanthropy. Containing a Review of the Position and Requirements of the Voluntary Charities, and an Exhaustive Record of Hospital Work for the Year. It will also be found to be the most Useful and Reliable Guide to British, Colonial, and American Hospitals, Dispensaries, Nursing and Convalescent Institutions and Asylums. Edited by HENRY C. BURDETT.

"Requires no more than the name of its editor to warrant that a subject of great and growing importance is dealt with in a thoroughly satisfactory manner."—*The Times*.

"The standard work of reference upon all points which relate to charities."—*Daily Telegraph*.

"Among the many works of reference which we owe to the indefatigable Mr. Henry C. Burdett, not the least useful is his 'Hospital and Charities Annual,' the volume of which for 1895 has been issued by the Scientific Press. It is a bulky volume of over 800 pages, and is crammed full of facts and figures. The first portion of the work is devoted to an examination of how much of the money given in charity is raised and spent, and contains many remarks which subscribers would do well to ponder. . . . The facts must have been got together at great labour and cost, and there is no work we know of that for fulness and variety of information on the subject with which it deals can at all compare with it."—*Westminster Gazette*.

Ready shortly. Third Edition. About 400 pp., profusely illustrated, Crown 8vo., price 10s. 6d.

### COTTAGE HOSPITALS, General and Convalescent. Their

Progress, Management, and Work. By HENRY C. BURDETT, Author of "Hospitals and Asylums of the World," "Pay Hospitals of the World," "Hospitals and the State," &c., &c.

The demand for copies of this useful and practical work exhausted the Second Edition soon after publication. The Third Edition is revised throughout and brought up to date, and profusely illustrated with new and original drawings.

"Contains all the information which could be required by anyone who undertakes to found or to manage a Cottage Hospital."—*British Medical Journal*.

Demy 8vo., profusely Illustrated with Specimen Tables, Index of Classification, Forms of Tender, &c., cloth extra, price 6s., post free.

### THE UNIFORM SYSTEM OF ACCOUNTS, AUDIT, AND

TENDERS, FOR HOSPITALS AND INSTITUTIONS, with Certain Checks upon Expenditure. Also the Index of Classification, compiled by a Committee of Hospital Secretaries, and adopted by a General Meeting of the same, January 18th, 1892. And certain Tender and other Forms for securing Economy. By HENRY C. BURDETT, Author of "Hospitals and Asylums of the World." "Burdett's Hospital Annual and Year-Book of Philanthropy."

A concise exposition of the Uniform System of Accounts, clearly explaining the nature and the method of keeping each of the books designed for the use of Hospitals and Institutions. Profusely illustrated with facsimile reproductions of pages from the Account Books, &c. It expounds practically the Uniform System, which is now being widely adopted as a means of saving labour and of bringing all systems of accounts into uniformity.

ACCOUNT BOOKS FOR INSTITUTIONS designed in accordance with

### THE UNIFORM SYSTEM OF ACCOUNTS FOR HOSPITALS

AND INSTITUTIONS. By HENRY C. BURDETT, Author of "Hospitals and Asylums of the World," "Burdett's Hospital Annual and Year-Book of Philanthropy," &c. Being a complete set of Royal, Foolscap, and Double Foolscap Account Books, ruled in accordance with the Uniform System adopted by the Metropolitan Hospital Sunday Fund. Designed and constructed for the convenience and assistance of Secretaries who present annual returns for participation in the Hospital Sunday Fund Grants.

These Books are ruled so that the various descriptions of Receipts and Expenditure, &c., &c., may be entered uniformly under the special headings and in the columns prepared for them. By their use the labour of Hospital Secretaries is reduced to a minimum, and the Accounts of all Public Institutions can be kept in a uniform manner. The adoption of a Uniform System of Accounts by Medical Charities has long been advocated, both by the author of this series and by Officials, Secretaries, and others connected with relief-giving Institutions.

Descriptive Price List post free on application.

Crown 8vo., cloth gilt, Illustrated, price 5s.

### HELPS IN SICKNESS AND TO HEALTH: Where to Go and What to Do. Being a Guide to Home Nursing and a Handbook to Health in the Habitation, the Nursery, the School-room and the Person, with a chapter on Pleasure and Health Resorts. By HENRY C. BURDETT, Author of "Hospitals and, Asylums of the World," "Pay Hospitals of the World," "Cottage Hospitals, General, and Convalescent," &c., &c.

"It would be difficult to find one which should be more welcome in a household than this unpretending but most useful book."—*The Times*.

"This book fills a gap in popular sanitary literature by providing within the compass of one volume, of very moderate size, a useful collection of facts not easily found elsewhere, unless a sanitary library be at hand."—*British Medical Journal*.

London: THE SCIENTIFIC PRESS (LIMITED), 428, STRAND, W.C.

### IMPORTANT NEW MEDICAL WORK.

PUBLISHED BY SUBSCRIPTION ONLY.

### TWENTIETH CENTURY PRACTICE

### An International Encyclopædia of MODERN MEDICAL SCIENCE

*BY LEADING AUTHORITIES OF EUROPE AND AMERICA.*

### Edited by THOMAS L. STEDMAN, M.D., New York City.

### IN TWENTY VOLUMES.

The work will be unique in the history of medical literature, not only by reason of its international character, but also because it will embody the testimony of experts only, each article having been entrusted to one recognised the world over as specially entitled to speak on that particular subject.

The aim of the work is, before all, a practical one. While the history of medicine is not ignored, and the sections on etiology and pathology will receive careful and thorough treatment, each author will nevertheless pay especial attention to questions of diagnosis and therapeutic management. In planning the work the needs and wants of the general practitioner have been constantly in view; it is for him that the treatises are written, and his approval will be the guarantee of the success of this monumental undertaking.

It is proposed that this work shall consist of twenty octavo volumes of between *seven hundred* and *eight hundred* pages each. New, bright-faced long primer type, cast especially for this purpose, illustrated wherever necessary from original designs, printed on fine paper, and bound in cloth extra, and in half morocco.

While the Publishers would be glad to avoid the immense detail, long experience has clearly demonstrated that a publication of this magnitude can be successfully issued when sold

### BY SUBSCRIPTION ONLY

for the entire series of TWENTY VOLUMES.

Prices at which the "Twentieth Century Practice" will be sold:

**In Cloth Extra, per vol., £1 1s. net; in Half Morocco, per vol., £1 10s. net,**

payable invariably on the delivery of the volumes at as nearly as possible regular intervals of three months.

It will be noted that by the plan of publication proposed this undertaking is to be completed by the close of the year **1899**, so that those who begin their subscription at once will, upon the most easy terms, find themselves the owners of what it is expected will be the most modern, most practical, most useful, and most authoritative work on the Practice of Medicine at the beginning of the Twentieth Century.

### CONTENTS OF THE VOLUMES OF "TWENTIETH CENTURY PRACTICE."

**Vol. I.—DISEASES OF THE UROPOIETIC SYSTEM.** [*Now ready.*
*Contributors*: Francis Delafield, New York; Reginald Harrison, London; G. Frank Lydston, Chicago; Howard A. Kelly, Baltimore; E. Hurry Fenwick, London.

**Vol. II.—NUTRITIVE DISORDERS.** [*Now ready.*
*Contributors*: Sir Dyce Duckworth, London; Carl von Noorden; Frankfort-on-the-Main; T. J. Maclagan, London; Henry M. Lyman, Chicago; Archibald E. Garrod, London; Dujardin-Beaumetz, Paris; Max J. Oertel, Munich.

**Vol. III.—POISONING AND MISCELLANEOUS.** [*Now ready.*
*Contributors*: Beaumont Small, Ottawa; James Stewart, Montreal; Norman Kerr, London; Albert L. Gihon, Washington; Georg von Liebig, Munich; James Hendrie Lloyd, Philadelphia; George F. Shrady, New York; Wm. T. Councilman, Boston.

**Vol. IV.—DISEASES OF THE CIRCULATORY SYSTEM.**
*Contributors*: James T. Whittaker, Cincinnati; A. Ernest Sansom, London; M. J. Oertel, Munich; George R. Murray, Newcastle-on-Tyne.

**Vol. V.—DISEASES OF THE SKIN.**
*Contributors*: L. Duncan Bulkley, New York; Arthur Van Harlingen, Philadelphia; H. Leloir, Lille; James Nevins Hyde, Chicago; H. Radcliffe Crocker, London; Wm. A. Hardaway, St. Louis; L. Brocq, Paris; John T. Bowen, Boston; D. W. Montgomery, San Francisco; M. Kaposi, Vienna.

**Vol. VI.—DISEASES OF THE RESPIRATORY ORGANS.**
*Contributors*: Prosser James, London; E. J. Moure, Bordeaux; Francke H. Bosworth, New York; Albert H. Buck, New York; Sir T. Grainger Stewart, Edinburgh; George A. Gibson, Edinburgh; Winslow Anderson, San Francisco.

**Vol. VII.—DISEASES OF THE RESPIRATORY ORGANS AND BLOOD AND FUNCTIONAL SEXUAL DISORDERS.**
*Contributors*: Th. von Jürgensen, Tübingen; Franz Reigel, Giessen; Dujardin-Beaumetz, Paris; E. Fletcher Ingals, Chicago; R. von Jaksch, Prague; S. Pozzi, Paris; Chas. W. Allen, New York; Jules Comby, Paris.

**Vol. VIII.—DISEASES OF THE DIGESTIVE ORGANS.**
*Contributors*: J. Mikuliscz, Breslau; Reginald H. Fitz, Boston; Max Einhorn, New York; Hobart A. Hare, Philadelphia; B. Farquhar Curtis, New York; Virgil P. Gibney, New York.

**Vol. IX.—DISEASES OF THE DIGESTIVE ORGANS.**
*Contributors*: C. A. Ewald, Berlin; M. Semmola, Naples; John B. Murphy, Chicago.

**Vol. X.—DISEASES OF THE NERVOUS SYSTEM.**
*Contributors*: B Sachs, New York; Charles L. Dana, New York; Ch. Féré, Paris; Charles K. Mills, Philadelphia; Sanger Brown, Chicago.

**Vol. XI.—DISEASES OF THE NERVOUS SYSTEM.**

*Contributors*: Paul J. Möbius, Leipsic; A. von Strüpnell, Erlangen; Azevedo Sodré, Rio de Janeiro; F. Raymond, Paris.

**Vol. XII.—MENTAL DISEASES, CHILDHOOD AND OLD AGE.**
*Contributors*: G. Fielding Blandford, London; Paul Sollier, Paris; Cesare Lombroso, Turin; James Cromby, Paris; Sir George M. Humphry, Cambridge; Ernest Hart, London; Emil Ponfick, Breslau.

**Vol. XIII.—INTRODUCTION TO INFECTIOUS DISEASES.**
*Contributors*: F. Loeffler, Griefswald; Victor C. Vaughan, Ann Arbor; Ch. Bouchard, Paris; Dawson Williams, London; Edwin Klebs, Strassburg.

**Vol. XIV. to XVIII.—INFECTIOUS DISEASES.**
*Contributors*: J. W. Moore, Dublin; P. Brouardel, Paris; Dillon Brown, New York; F. Forchheimer, Cincinnati; Dawson Williams, London; Jules Comby, Paris; Joseph O'Dwyer, New York; A. Jacobi, New York; D. Finkler, Bonn; Sir Joseph Fayrer, London; David Bruce, British Army; N. Gamaleïa, St. Petersburg; I. Boas, Berlin; Emil Ponfick, Breslau; Thomas J. Moore, Richmond; Edwin Klebs, Strassburg; Edoardo Liceaga, Mexico; George M. Sternberg, U.S. Army; P. S. Connor, Cincinnati; Th. Rumpf, Hamburg; Ettore Marchiafava, Rome; Azevedo Sodré, Rio de Janeiro; Victor Babes, Bucharest; J. McFadden Gaston, Atlanta; E. Ziegler, Freiburg i. Br.; Hunter McGuire, Richmond.

**Vol. XIX.—TUBERCULOSIS AND LEPROSY.**
*Contributors*: William H. Welch, Baltimore; E. Maragliano, Genoa; G. Armauer Hansen, Bergen, Norway.

**Vol. XX.—SYPHILIS AND GENERAL INDEX.**
*Contributors*: Jonathan Hutchinson, London; Eduard Lang, Vienna.

***** Send for Prospectus, 20 Pages, Giving Full Particulars and Specimens of the Illustrations and Text Pages.**

London: SAMPSON LOW, MARSTON, and COMPANY (Limited), St. Dunstan's House, Fetter Lane, Fleet Street, E.

### CASSELL & COMPANY'S ANNOUNCEMENTS.

### NEW AND RECENT VOLUMES.

**A NEW AND ORIGINAL WORK ON SURGERY.**

*NOW READY.* *The First Volume of*

### A SYSTEM OF SURGERY.

BY VARIOUS AUTHORS.

Edited by FREDERICK TREVES, F.R.C.S.

*With Two Coloured Plates and Several Hundred Original Woodcut Illustrations by CHARLES BERJEAU, F.L.S., and others.*

To be completed in Two Volumes, price 48s. Vol. II. is in active preparation.

NOW IN THE PRESS. PRICE 21s.

### INJURIES AND DISEASES OF THE GENITAL AND URINARY ORGANS.

BY

**HENRY MORRIS, M.A., M.B.Lond., F.R.C.S.Eng.,**

Surgeon to, and Lecturer on Surgery at, the Middlesex Hospital; Member of the Council, and of the Court of Examiners, of the Royal College of Surgeons (England); Examiner in Surgery in the University of London.

*PROFUSELY ILLUSTRATED.*

DISEASES OF THE JOINTS AND SPINE. By HOWARD MARSH, F.R.C.S. 12s. 6d.

DISEASES OF THE EAR. By A. Marmaduke SHEILD, M.B.Cantab., F.R.C.S.Eng. 10s. 6d.

DISEASES OF THE SKIN. By Malcolm Morris, F.R.C.S.Ed. With 8 Chromo-lithographs and 17 Woodcuts. 10s. 6d.

DIFFICULT LABOUR. A Guide to its Management for Students and Practitioners. By G. ERNEST HERMAN, M.B. With 162 Illustrations. 12s. 6d.

A MANUAL OF MEDICAL TREATMENT, or, CLINICAL THERAPEUTICS. By I. BURNEY YEO, M.D. Fourth Edition, revised. Two vols. 21s.

A MANUAL OF OPERATIVE SURGERY. By FREDERICK TREVES, F.R.C.S. With 422 Illustrations. Third Thousand. Two vols. 42s.

SURGICAL DISEASES OF THE OVARIES and FALLOPIAN TUBES. By J. BLAND SUTTON, F.R.C.S. With 118 Engravings and 5 Coloured Plates. 12s. 6d.

DISEASES OF THE RECTUM AND ANUS. By CHARLES B. BALL, M.Ch.Dublin. With Chromo Plates. 9s.

TUMOURS, INNOCENT AND MALIGNANT. By J BLAND SUTTON, F.R.C.S. With 250 Engravings and 9 Plates. 21s.

### MANUALS FOR STUDENTS OF MEDICINE.

| | | |
|---|---|---|
| A MANUAL OF CHEMISTRY, INORGANIC AND ORGANIC. With numerous Engravings... ... ... | By ARTHUR P. LUFF, M.D., B.Sc.Lond., M.R.C.P. ... ... ... ... ... ... | 7s. 6d. |
| THE STUDENT'S HANDBOOK OF SURGICAL OPERATIONS. With 94 Illustrations ... ... ... ... | By FREDERICK TREVES, F.R.C.S. ... | 7s. 6d. |
| SURGICAL APPLIED ANATOMY. Fifteenth Thousand | By FREDERICK TREVES, F.R.C.S. ... | 7s. 6d. |
| COMPARATIVE ANATOMY AND PHYSIOLOGY ... | By F. JEFFREY BELL, M.A. ... ... | 7s. 6d. |
| PHYSIOLOGICAL PHYSICS ... ... ... ... ... ... | By J. McGREGOR ROBERTSON, M.A.... | 7s. 6d. |
| HUMAN PHYSIOLOGY. Fourth and Enlarged Edition | By HENRY POWER, M.B. ... ... | 7s. 6d. |
| MATERIA MEDICA AND THERAPEUTICS ... ... | By J. MITCHELL BRUCE, M.D. ... ... | 7s. 6d. |
| ELEMENTS OF HISTOLOGY. New and Enlarged Edition | By E. KLEIN, M.D. ... ... ... | 7s 6d. |
| FIRST LINES IN MIDWIFERY: a Guide to Attendance on Natural Labour ... ... ... ... ... ... | By G. E. HERMAN, M.B. ... ... ... | 5s. 0d. |
| HYGIENE AND PUBLIC HEALTH... ... ... ... | By B. ARTHUR WHITELEGGE, M.D.... | 7s. 6d. |
| A MANUAL OF SURGERY. (Three Vols.) ... ... | Edited by F. TREVES, F.R.C.S. ... each | 7s. 6d. |
| SURGICAL DIAGNOSIS: a Manual for the Wards ... | By A. PEARCE GOULD, M.B. ... ... | 7s. 6d. |
| CLINICAL CHEMISTRY ... ... ... ... ... | By CHARLES H. RALFE, M.D. ... ... | 5s. 0d. |
| SURGICAL PATHOLOGY. Fourth Edition, Rewritten and Enlarged ... ... ... ... ... ... | By A. J. PEPPER, M.B. ... ... ... | 8s. 6d. |

### CLINICAL MANUALS FOR STUDENTS AND PRACTITIONERS OF MEDICINE.

| | | |
|---|---|---|
| ON GALL-STONES AND THEIR TREATMENT... ... | By Prof A. W. MAYO ROBSON, F.R.C.S. | 9s. 0d. |
| THE PULSE ... ... ... ... ... ... | By Sir W. H. BROADBENT, Bart., M.D. | 9s. 0d. |
| FOOD IN HEALTH AND DISEASE ... ... ... | By I. BURNEY YEO, M.D. ... ... | 9s. 0d. |
| DISEASES OF THE BREAST... ... ... ... | By THOMAS BRYANT, F.R.C.S. ... | 9s. 0d. |
| OPHTHALMIC SURGERY ... ... ... ... ... | By R. BRUDENELL CARTER, F.R.C.S., and W. ADAMS FROST, F.R.C.S. ... | 9s. 0d. |
| SYPHILIS ... ... ... ... ... ... ... ... | By JONATHAN HUTCHINSON, F.R.C.S. | 9s. 0d. |
| FRACTURES AND DISLOCATIONS ... ... ... | By T. PICKERING PICK, F.R.C.S. ... | 9s. 0d. |
| SURGICAL DISEASES OF THE KIDNEY ... ... | By H. MORRIS, M.B. ... ... ... ... | 9s. 0d. |
| INSANITY AND ALLIED NEUROSES ... ... ... | By G. H. SAVAGE, M.D. ... ... ... | 9s. 0d. |
| INTESTINAL OBSTRUCTION ... ... ... ... | By FREDERICK TREVES, F.R.C.S. ... | 9s. 0d. |
| DISEASES OF THE TONGUE ... ... ... ... | By H. T. BUTLIN, F.R.C.S. ... ... | 9s. 0d. |
| SURGICAL DISEASES OF CHILDREN ... ... ... | By E. OWEN, M.B., F.R.C.S. ... ... | 9s. 0d. |

*** *A Catalogue of Cassell & Company's MEDICAL and CLINICAL MANUALS will be sent post free on application.*

CASSELL & COMPANY, LIMITED, LUDGATE HILL, LONDON.

### "THE HOSPITAL,"
AN INDEPENDENT JOURNAL OF
**THE MEDICAL SCIENCES AND HOSPITAL ADMINISTRATION,**
**Now ready, MONTHLY PART FOR OCTOBER, price 6d.,**
Containing interesting Articles on :—

The Respiration of Plants.
The Study of Man.
English Doctors Abroad.
On Sight-seeing.
On Modern Progress in Ophthalmic Medicine and Surgery. By R. B. Carter, F.R.C.S.
The Symptoms of Aneurisms. By A. Pearce Gould, F.R.C.S.
Drugs and Drink.
Medical Progress and Hospital Clinics.
Chloroform Fatalities.—III. By Sir B. W. Richardson, M.D., F.R.S.
Social Problems.
New Appliances & Things Medical.
Annotations on Various Subjects.
The Institutional Workshop.
The Book World of Medicine and Science.

Publishing Offices: 428, Strand, London, W.C.

### STANDARD TEXT-BOOKS FOR NURSES.

THE NURSES' DICTIONARY OF MEDICAL TERMS AND NURSING TREATMENT. By HONNOR MORTEN. 2nd Edition. Demy 16mo. Cloth 2s., leather 2s. 6d.

THEORY AND PRACTICE OF NURSING. By PERCY G. LEWIS, M.D. Crown 8vo. Cloth, 400 pp. Profusely illustrated. 3s. 6d.

NURSING. By ISABEL ADAMS HAMPTON. 2nd Edition. Crown 8vo. Cloth, 500 pp. 7s. 6d. net.

THE MOTHER'S HELP AND GUIDE TO THE DOMESTIC MANAGEMENT OF CHILDREN. By P. MURRAY BRAIDWOOD, M.D. Crown 8vo. Cloth gilt. 2s. 6d.

ART OF MASSAGE. By A. CREIGHTON HALE. Demy 8vo. Cloth. Illustrated. 6s.

SURGICAL WARD-WORK AND NURSING. By ALEXANDER MILES, M.D. (Edin.), C.M., F.R.C.S.E. Demy 8vo. Cloth. With numerous illustrations. 3s. 6d.

HOSPITAL SISTERS AND THEIR DUTIES. By EVA C. E. LUCKES. 3rd Edition. Crown 8vo. Cloth. 2s. 6d.

INFANT FEEDING BY ARTIFICIAL MEANS. By S. H. SADLER. Crown 8vo. Cloth. Illustrated. 5s.

OPHTHALMIC NURSING. By SIDNEY STEPHENSON, M.B., F.R.C.S.E. Crown 8vo. Cloth. Illustrated. 3s. 6d.

ART OF FEEDING THE INVALID. By a Medical Practitioner and a Lady Professor of Cookery. Demy 8vo. Cloth, 260 pp. 3s. 6d.

OUTLINES OF INSANITY. By FRANCIS H. WALMSLEY, M.D. Demy 8vo. Cloth. 3s. 6d. Popular Edition, stitched, 1s. 6d.

MENTAL NURSING. By WILLIAM HARDING, M.D. 2nd Edition. Demy 8vo. Cloth. 2s. 6d.

"THE HOSPITAL" NURSES' CASE BOOK. For use in hospital as well as private nursing. Demy 16mo. (for apron pocket). 6d. each, or 4s. 6d. per doz.

London THE SCIENTIFIC PRESS (LIMITED), 428, STRAND, LONDON, W.C.

### ROYAL NATIONAL PENSION FUND FOR NURSES.

ROYAL NATIONAL PENSION FUND FOR NURSES,

Ready next week. In handsome cloth binding. Price 6s.

### VOL. XVIII. OF "THE HOSPITAL,"

**APRIL to SEPTEMBER, 1895.**

**ALSO INDEX TO VOL. XVIII., ENDING SEPTEMBER 28th, 1895.**

Price 2½d., post free.

Publishing Offices, 428, Strand, London, W.C.

